# Prenatal and intrapartum antibiotic exposure and childhood infections: considerations and complexities

**DOI:** 10.1016/j.lana.2025.101368

**Published:** 2026-01-07

**Authors:** Isobel M.F. Todd, David P. Burgner, Maria C. Magnus, Lars Henning Pedersen, Jessica E. Miller

**Affiliations:** a Murdoch Children's Research Institute, Parkville, Victoria, Australia; b Faculty of Medicine, Dentistry and Health Sciences, The University of Melbourne, Parkville, Victoria, Australia; c Department of Infectious Diseases, Royal Children's Hospital, Parkville, Victoria, Australia; d Centre for Fertility and Health, Norwegian Institute of Public Health, Oslo, Norway; e Clinical Medicine, Aarhus University, Denmark; f Department of Obstetrics and Gynecology, Aarhus University Hospital, Denmark; g WHO Collaborating Centre for Reference and Research on Influenza, The Peter Doherty Institute of Infection and Immunity, Parkville, Victoria, Australia

Coggins et al.^1^ reported a lack of association between intravenous perinatal and early postnatal antibiotics and subsequent childhood infections. They contrast their findings with those from other studies of pregnancy and intrapartum antibiotic use, which show dose–response associations with later infection, and in which increased risk is thought to result from immunological consequences of antibiotic-associated dysbiosis.^2^, ^3^, ^4^ They conclude that their findings differ from these previous studies. However, their adjusted estimate of 1.16 (95% CI 0.95–1.51) is a similar magnitude to previous studies (quoted estimates ranging between 1.10 and 1.34); the difference is that their confidence interval includes the null.

The current study only investigated intravenous antibiotics around birth, whereas previous studies mainly included oral antibiotics throughout pregnancy, a more common exposure of longer duration. The route of antibiotic administration may impact the intestinal microbiome differently, including the rate of dysbiosis resolution.^5^ Coggins et al. did not consider prior antibiotic exposure, which occurs in approximately 20% of pregnancies^2^^,^^3^ and may modify the effects of subsequent perinatal intravenous antibiotics on the microbiome.

A major challenge when investigating antibiotics as an exposure is confounding by indication, which was not explored in detail by Coggins et al., beyond multivariable adjustment for mode of birth, group B streptococcal colonisation and chorioamnionitis. In addition, they did not distinguish vertically acquired (early-onset) from late-onset infections, which may underestimate the association.

Future studies should consider oral antibiotics during pregnancy and intravenous perinatal antibiotics, as each may contribute differently to associations, and should explore confounding by indication in detail.

## Declaration of interests

The authors declare no conflicts of interest.
